# Supplementary material for: Superfolder mTurquoise2ox optimized for the bacterial periplasm allows high efficiency in vivo FRET of cell division antibiotic targets
Source: Mol Microbiol. 2019 Feb 28;111(4):1025–38. doi: 10.1111/mmi.14206 (PMC6850650; doi:10.1111/mmi.14206)
Supplement: Supplementary file 1 [file MMI-111-1025-s001.docx]

Superfolder mTurquoise2^ox^ optimized for the bacterial periplasm allows high efficiency *in vivo* FRET of cell division antibiotic targets.

Nils Y. Meiresonne^1^, Elisa Consoli^1a^, Laureen M. Y. Mertens^1a^, Anna Chertkova^2^, Joachim Goedhart^2^ and Tanneke den Blaauwen^1*^

^1^Bacterial Cell Biology & Physiology, Swammerdam Institute for Life Sciences, University of Amsterdam, Science park 904, 1098 XH Amsterdam, The Netherlands.

^2^Molecular Cytology and van Leeuwenhoek Centre for Advanced Microscopy, Swammerdam Institute for Life Sciences, University of Amsterdam, Science park 904, 1098 XH Amsterdam, The Netherlands.

*corresponding author

## Table S1

Förster radii of FRET pairs of the mNG donor with cysteine-less orange and red FPs or with mNG as an acceptor to the new sfTq2^ox^ donor (**described in main text**).

| mNG + FP | R_0_ (nm)* |
| --- | --- |
| mOrange | 5.67 |
| mStrawberry | 5.93 |
| mCherry | 5.51 |
| mScarlet | 5.95 |
| mScarlet-I | 5.97 |
| mScarlet-H | 6.02 |
| sfTq2^ox^ | 5.96 |

Biophysical properties used to calculate the R_0_ values can be found in Cranfill *et al* 2016 and Bindels *et al* 2017 (Cranfill *et al.*, 2016; Bindels *et al.*, 2016). *R_0_ calculated with refractive index of 1.4 for cellular environments.

## Figure S1 – Toxicity and fluorescence of expressing cysteine-less FPs in the periplasm.

a) Expression of DsbA^SS^-FP-PBP5 was induced at a concentration range of IPTG in LMC500 and growth and fluorescence were monitored in TY at 37 °C in a plate reader. All constructs resulted in toxicity at high induction levels. Low induction was accepted and resulted in fluorescence over time. Note the differences in scale for the fluorescent signals. b) Periplasmic sfGFP and mNG from overnight cultures. c) Periplasmic mOr, mOr2, mSc-H, mSc, mSc-I, mStr and mCh from overnight cultures. A negative control consisted of cells harboring a plasmid without any FP (EV). All scale bars represent 2 µm.

## Figure S2 – mFruits in the periplasm.

a) LMC500 carrying DsbA^ss^-FP-PBP5 plasmids for periplasmic expression of selected mFruit FPs were grown as flask cultures in TY at 37 °C and induced with 15 µM IPTG (arrow). Induction did not alter growth rates compared to a control carrying an empty plasmid (EV). b) Fluorescence images of living, fixed and fixed and matured cells showed varying levels of periplasmic fluorescence. Note that the grayscales were set for automatically visualizing any signal. The scale bar represents 2 µm. c) Quantification of fluorescence signals from all samples revealing strong periplasmic signals for mCherry. The respective number of cells analyzed for the living, fixed or fixed and matured samples were; EV 368, 781 and 754, mOr 604, 738 and 1258, mOr2 532, 736 and 781, mStr 784, 836 and 1333, and mCh 954, 1255 and 760. The error bars at the mean indicate the 95 % confidence interval.

# Figure S3 - Residue 145 of mTq2 is important for chromophore formation.

LMC500 was grown in TY and expression of mTq2-PBP5 or sfTq2-PBP5 with or without the additional Y145F mutation, was induced with 15 µM IPTG. a) Living, fixed and fixed and matured samples were imaged. The scale bar represents 2 µm. b) Quantification of the signals shows that Y145F does not reveal an improvement of the periplasmic mTq2 signal and shows a negative impact on periplasmic sfTq2 fluorescence. Between 500 and 1300 cells were analyzed for all samples except EV alive (n = 217) and EV matured (n = 373). The error bars at the mean represents the 95 % confidence interval.

# Supplementary text 1: Cysteine-less sfTq2 variants

Site-directed mutagenesis of the sfTq2 native cysteines at positions 48 and 70 resulted in much stronger fluorescence signals from the periplasm caused by a higher protein yield and reduced expression toxicity (**main text**). sfTq2^C70V^ was found to be the brightest variant with the fastest folding/maturation rates and low toxicity levels. To screen for more variation in periplasmic fluorescence, and possibly a better variant, targeted random mutagenesis was performed on residues 48 and 70 of sfTq2, on residues 70 of sfTq2^C48S^ and on residue 48 of sfTq2^C70V^. NNK/MNN primers encoding degenerate codons for all 20 possible amino acids were used (**Table S4**). The resulting mutagenesis colonies were spotted on TY agar containing 10 µM IPTG and incubated overnight at 37 °C. The new colonies were observed using a macroscopic fluorescence set up and the brightest were selected for sequence analysis.

The random mutations introduced at position 48 of sfTq2 or sfTq2^C70V^ were mainly serines and cysteines, respectively. The random mutations of position 70 introduced in sfTq2^C48S^ resulted in WT cysteine, methionine or valine, which were already made by site-directed mutagenesis. This suggest that the serine at position 48 instead of cysteine may be the only tolerated residue substitution for periplasmic FP folding and chromophore maturation and additional mutations of C70 could decreases these positive effects.

Random mutagenesis of C70 in sfTq2 showed more variation and yielded Alanine, Proline, Serine or Threonine, which are relatively small amino acids. Direct comparison experiments showed that sfTq2^C70V^ has at least equally reduced expression toxicity and provides the fastest and brightest fluorescence in the periplasm of all other sfTq2-variants (**Fig. S6 and S7**). These favorable properties were confirmed with additional periplasmic fusion proteins associated with the IM (NlpA) or the OM (OmpA and LpoB) (**Figs. S10-12**).

## Figure S4 – Site-directed sfTq2 cysteine mutants in the periplasm.

Cysteine-replaced sfTq2 variants perform better in the periplasm. a) Periplasmic expression of sfTq2-PBP5 variants in LMC500 grown in rich medium at 37°C at relatively non-toxic induction conditions results in large differences in cyan fluorescence. b) Microscopy of LMC500 grown in rich medium induced with 15 µM IPTG shows strong fluorescence for the sfTq2-PBP5 variants in the periplasm. For comparison the grayscale of all photographs is the same (80-7000) and the scale bar represents 2 µm. c) Quantification of the images confirms bright fluorescence signals from the single C48S and C70V sfTq2 variants. The error bars at the mean represents the 95 % confidence interval.

## Figure S5 – Random mutagenesis of the native cysteines in sfTq2 did not result in a better variant compared to sfTq2^C70V^.

LMC500 was grown in rich medium at 37 °C in a plate reader expressing single cysteine mutants for position 48 or 70 acquired by directed or random mutagenesis at non-toxic induction levels (8 µM IPTG). The expression vector was sfTq2-PBP5 with the indicated single mutations, mTq2-PBP5 served as a control. a) Comparison of growth and fluorescence over time for all single sfTq2 variants in the periplasm. b) Comparison of the C70A, C70T and C70V sfTq2 variants in the periplasm showing similar growth but superior fluorescence for C70V.

## Figure S6 – mTq2 does not benefit from the cysteine mutations in terms of fluorescence.

a) LMC500 carrying periplasmic mTq2 cysteine variants on plasmid as PBP5-mTq2^xx^ were grown in the plate reader in rich medium at 37°C at a concentration range of IPTG inducer while monitoring growth and fluorescence. At intermediate induction conditions there was a clear benefit in growth for the cysteine replaced variants. In terms of fluorescence, sfTq2 was the only FP capable of showing detectable periplasmic signals. b) The same groups were grown as flask cultures in TY at 37 °C and expression was induced with 15 µM IPTG for at least 2 MDs. Living, fixed and fixed and matured samples were imaged and the fluorescence intensities were quantified revealing no improved fluorescence of either of the mTq2 cysteine mutant variants compared to mTq2. Between 500 and 1000 cells were analyzed for each of the groups. The error bars at the mean represent the 95% confidence interval.

## Figure S7– sfGFP fluorescence does not benefit from the C70V mutation in terms of fluorescence.

LMC500 carrying PBP5-sfGFP or PBP5-sfGFP^70V^ plasmids was grown as flask cultures in rich medium at 37 °C and periplasmic expression was induced with 15 µM IPTG for at least 2 MDs. Cells carrying the plasmid not coding for a fusion protein served as a control (EV). a) Living, fixed or fixed and matured samples were imaged by fluorescence microscopy. The photographs are presented at the same gray values for comparison and the scale bar represents 2 µm. b) The fluorescence intensities were quantified, revealing no major differences between periplasmic sfGFP and its C70V variant. Between 400 and 900 cells were analyzed for each of the groups. The error bars at the mean represent the 95% confidence interval.

## Figure S8 – Quantification of sfTq2 and sfTq2^ox^ as fusion to periplasmic IM-associated NlpA.

LMC500 carrying NlpA-sfTq2 or NlpA-sfTq2^ox^ plasmids were grown as flask cultures in rich medium at 37 °C and periplasmic expression was induced with 10 µM IPTG for at least 2 MDs. Cells carrying the plasmid not coding for a fusion protein served as a control (EV). a) Samples of living, fixed and fixed and matured cells were imaged. The photographs are presented at the same gray values for comparison and the scale bar represents 2 µm. b) At least 500 cells per group were quantified, except for EV matured (n = 442). The error bars at the mean represent the 95 % confidence interval.

## Figure S9 – Quantification of sfTq2 and sfTq2^ox^ as fusion to periplasmic OM-inserted OmpA177.

LMC500 carrying OmpA177-sfTq2 or OmpA177-sfTq2^ox^ plasmids were grown as flask cultures in rich medium at 37 °C and periplasmic expression was induced with 15 µM IPTG for at least 2 MDs. Cells carrying the plasmid not coding for a fusion protein served as a control (EV). a) Samples of living, fixed and fixed and matured cells were imaged. The photographs are presented at the same gray values for comparison and the scale bar represents 2 µm. b) Fluorescence intensities were quantified. At least 500 cells per group were quantified, except for sfTq2^ox^ alive (n = 472) and sfTq2 fixed (n = 417). The error bars at the mean represent the 95 % confidence interval.

## Figure S10 – Quantification of sfTq2 and sfTq2^ox^ as fusion to periplasmic OM-associated LpoB.

LMC500 carrying periplasmic LpoB-sfTq2 or LpoB-sfTq2^ox^ plasmids were grown as flask cultures in rich medium at 37 °C and induced with 15 µM IPTG for at least 2 MDs. Cells carrying the base plasmid not coding for a fusion protein served as a control (EV). a) Samples of living, fixed and fixed and matured cells were imaged. The photographs are presented at the same gray values for comparison and the scale bar represents 2 µm. b) Fluorescence intensities were quantified. At least 500 cells per group were quantified, except for alive samples EV (n = 368), sfTq2 (n = 466) and sfTq2^ox^ fixed (n = 482). The error bars at the mean represent the 95 % confidence interval.


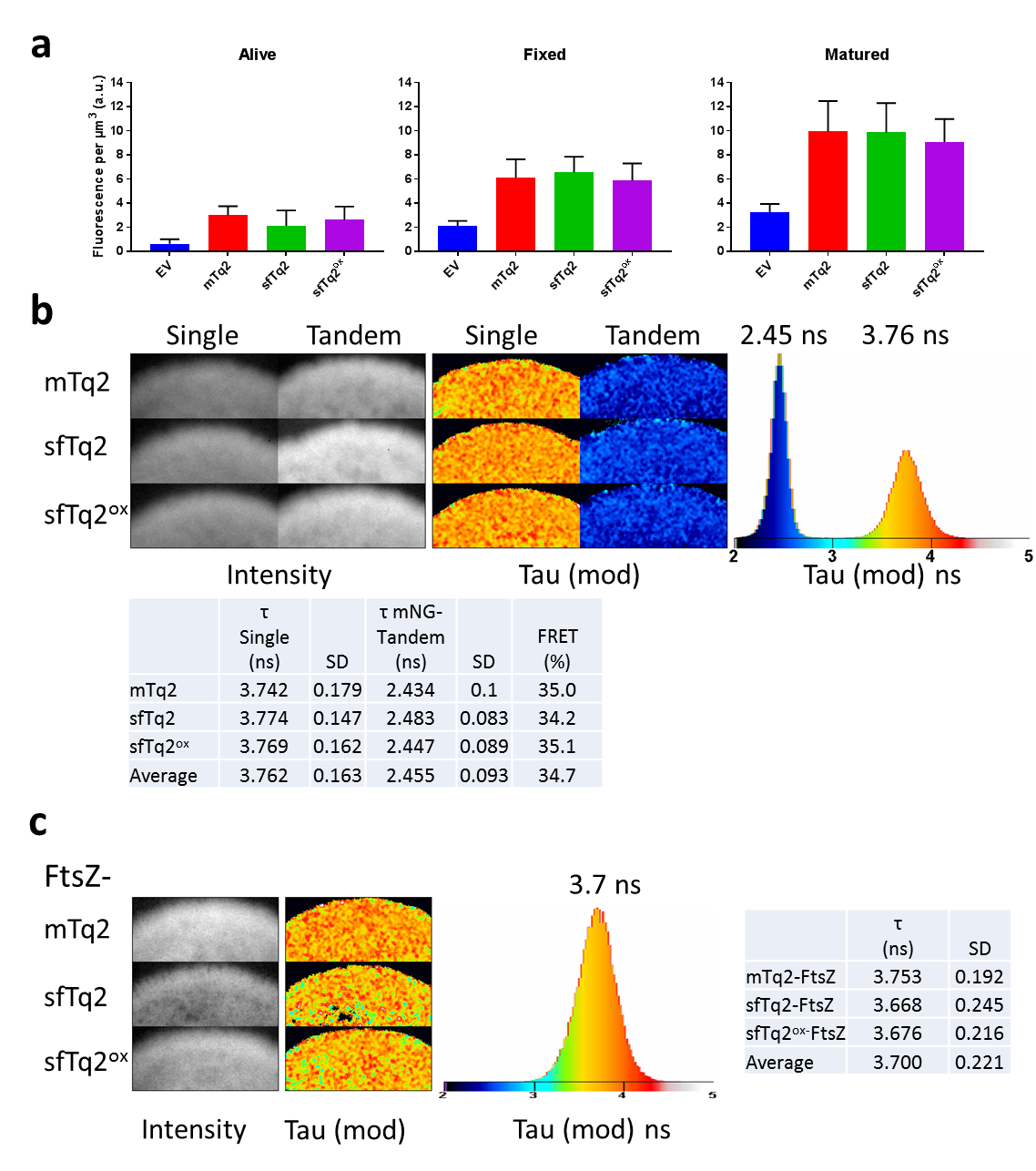


# Figure S11 - sfTq2^ox^ comes without a trade-off

a) LMC500 harboring free-floating cytoplasmic mTq2, sfTq2 or sfTq2^ox^ expressed from plasmid was grown as flask cultures in rich medium at 37°C and expression was induced with 50 µM IPTG for at least 2 MDs. Living, fixed and fixed and matured samples were imaged and quantified. The fluorescence intensities of all cytosolic mTq2 variants were similar. Between 200 and 1055 cells were analyzed for the living samples. At least 600 cells were analyzed for the fixed and the fixed and matured samples. The error bars at the mean indicate the standard deviation. b) Fluorescence lifetimes were detected of LMC500 colonies harboring free-floating cytoplasmic mTq2, sfTq2 or sfTq2^ox^ and their respective mNG tandem on TY agar plates containing 10 µM IPTG. The single FPs had a similar average lifetime of 3.76 ns. The tandem fusions were also matching in lifetime but had on average a shorter lifetime of 2.45 ns. This corresponds to a FRET efficiency of 35 % for the tandems regardless of the mTq2 variant used. c) Fluorescence lifetimes were detected of LMC500 colonies harboring cytoplasmic mTq2, sfTq2 or sfTq2^ox^ as N-terminal fusions to FtsZ on the plates described above. This revealed similar lifetimes with an average of 3.7 ns.


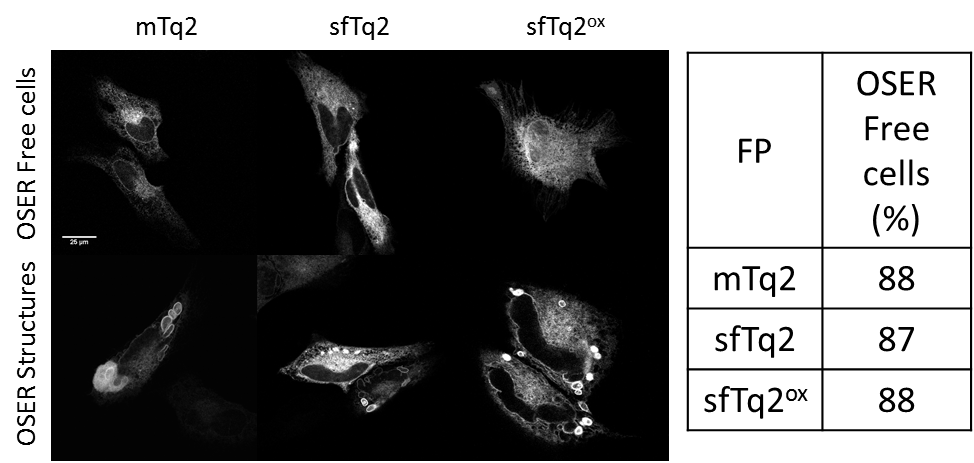


# Figure S12 – OSER assay reveals the similar multimerization propensity for mTq2, sfTq2 and sfTq2^ox^.

The top row shows cells free of OSER structures and the bottom row shows cells with OSER structures. The scalebar indicates 25 µm. One-hundred cells of each group were checked and resulted in a similar percentage of OSER free cells between the three mTq2 variants.





# Figure S13 –Western blots show no degradation products of periplasmic constructs.

LMC500 carrying indicated mTq2 variants on plasmid were grown as flask cultures in rich medium at 37 °C and induced with 15 µM IPTG for at least 2 MDs after which samples were taken for western blotting with αGFP (**materials and methods**). The same cultures were used for quantifying fluorescent signals from microscopy images shown in the **main text and supplementary information**. Samples were made with and without DTT reducing agent to show possible oligomerization under oxidative conditions. This was observed mildly for sfTq2^C48S^-PBP5 only (**Fig. 3 main text**). The numbers beside the blots indicate molecular mass markers (kDa).

# Figure S14 – Overview of representative unmixing of all mNG-mTq2 variant FRET samples of table 1 measured in the fluorometer.

The title above the graphs applies to emission graphs of the donor excitation and the acceptor excitation beneath it. The black dots show the measured spectrum and the red line the calculated spectrum. These are dissected into a spectrum for the background (gray), mTq2 (blue), mNG (green) and the sensitized emission (orange). Residual signal that could not be accounted for is plotted as unmixing difference and serves as a measure of quality. Unmixings using mTq2 or sfTq2 are indicated, the others are with sfTq2^ox^.

# Table S2 – E*f*_A_ values calculated for FRET samples measured with the plate reader set-up.

| Cytoplasmic FRET | Donor FP to mNG | Fusion | *Ef_A_* (%) | SD | Repeats |
| --- | --- | --- | --- | --- | --- |
| **Positive control tandems** | | | | | |
| mNG-mTq2 | mTq2 | Free floating | 59.0 | 7.9 | 4 |
| mNG-sfTq2 | sfTq2 | Free floating | 65.1 | 6.3 | 12 |
| mNG-sfTq2^C70V^ | sfTq2^ox^ | Free floating | 65.3 | 2.6 | 8 |
|  |  |  |  |  |  |
| Periplasmic FRET |  |  |  |  |  |
| **Positive control tandems** | | | | | |
| mNG-sfTq2 | sfTq2 | OmpA177 | 21.0 | 3.6 | 7 |
| mNG-sfTq2^C48S^ | C48S | OmpA177 | 35.6 | 4.3 | 8 |
| mNG-sfTq2^C70V^ | sfTq2^ox^ | OmpA177 | 37.9 | 4.7 | 13 |
| mNG-sfTq2^C48S-C70V^ | C48S-C70V | OmpA177 | 40.6 | 1.8 | 8 |
| mNG-sfTq2^C70V^ | sfTq2^ox^ | OmpA177* | 42.9 | 3.4 | 10 |
| mNG-sfTq2^C70V^ | sfTq2^ox^ | LpoB73 | 36.7 | 1.9 | 5 |
| mNG-sfTq2^C70V^ | sfTq2^ox^ | NlpA^ss^ | 41.4 | 1.6 | 7 |
|  |  |  |  |  |  |
| **Negative controls** | | | | | |
| mNG-sfTq2 (IM-OM) | sfTq2 | PBP5-OmpA177 | -0.0 | 3.0 | 8 |
| mNG-sfTq2^C70V^ (IM-OM) | sfTq2^ox^ | PBP5-OmpA177 | -1.1 | 3.2 | 16 |
| mNG-sfTq2^C70V^ (IM-IM) | sfTq2^ox^ | NlpA^ss^-FtsB | -1.8 | 7.4 | 10 |
|  |  |  |  |  |  |
| **Biological interactions** | | | | | |
| PBP5-PBP5 | sfTq2 | IM-IM | 2.7 | 1.0 | 3 |
| PBP5^S44G^-PBP5^S44G^ | sfTq2 | IM-IM | 9.5 | 2.6 | 3 |
| FtsB-FtsL | sfTq2^ox^ | IM-IM | 16.2 | 3.2 | 9 |
| FtsL-FtsB | sfTq2^ox^ | IM-IM | 16.0 | 3.7 | 8 |

^SS^ is signal sequence, LpoB73 indicates residue 1-73 of LpoB, OmpA177 indicates residue 1-177 of OmpA, * indicates a different linker between mNG and sfTq2^ox^ (EF instead of EL, due to different cloning). Representative unmixing data is shown in **Fig. S15**.

# Figure S15 – Overview of representative unmixing of all mNG-mTq2 FRET samples shown in table S2 measured in the plate reader.

The title above the graphs applies to emission graphs of the donor excitation and the acceptor excitation beneath it. The black dots show the measured spectrum and the red line the calculated spectrum. These are dissected into a spectrum for the background (gray), mTq2 (blue), mNG (green) and the sensitized emission (orange). Residual signal that could not be accounted for is plotted as unmixing difference and serves as a measure of quality. Unmixings with mTq2 or sfTq2 are indicated, the others are with sfTq2^ox^.

# Table S3 – Plasmids used and cloning strategies

| **Short name** | **Long name** | **Information** | **Ref.** | **Vector backbone** | **primers** | **RE1** | **RE2** | **Insert** | **Template** | **Primers** | **RE1** | **RE2** |
| --- | --- | --- | --- | --- | --- | --- | --- | --- | --- | --- | --- | --- |
| **pTHV037** |  | Basic expression vector ptrc99A derived, differing from in its with different -35 promotor region (TTGACA-TTTACA). pBR322 origin, ampR | (Den Blaauwen *et al.*, 2003) |  |  |  |  |  |  |  |  |  |
| **pSAV057** |  | Basic expression vector ptrc99A derived, with different -35 promotor Region (TTGACA-TTTACA) p15 origin, catR | (Alexeeva *et al.*, 2010) |  |  |  |  |  |  |  |  |  |
|  | **Cytoplasmic Expression constructs** |  |  |  |  |  |  |  |  |  |  |  |
| **pNM001** | pSAV057-mNG^EC^ | Used for cloning | (Meiresonne *et al.*, 2017) |  |  |  |  |  |  |  |  |  |
| **pNM002** | pSAV057-mNG^EC^-mCh | Used for cloning | (Meiresonne *et al.*, 2017) |  |  |  |  |  |  |  |  |  |
| **pNM040** | pSAV057-mTq2 | Cytoplasmic mTq2 reference | This work | pNM002 |  | NcoI | BsrGI | mTq2 | pNM059 | 234 + 86 | NcoI | BsrGI |
| **pNM041** | pSAV057-sfTq2 | Cytoplasmic sfTq2 reference | This work |  |  |  |  |  |  |  |  |  |
| **pNM042** | pSAV057-sfTq2^C70V^ | Cytoplasmic sfTq2^ox^ reference | This work |  |  |  |  |  |  |  |  |  |
| **pNM043** | pSAV057-mNG-mTq2 | Cytoplasmic mTq2 tandem | This work | pNM002 |  | EcoRI | HindIII | mTq2 | pNM059 | 39 + 86 | MfeI | HindIII |
| **pNM044** | pSAV057-mNG-sfTq2 | Cytoplasmic sfTq2 tandem | This work |  |  |  |  |  |  |  |  |  |
| **pNM045** | pSAV057-mNG-sfTq2^C70V^ | Cytoplasmic mNG^EC^sfTq2^ox^ tandem | This work | pNM002 |  | EcoRI | HindIII | sfTq2-C70V | pNM077 | 39 + 86 | MfeI | HindIII |
| **pSAV073** |  | Used for cloning | (Alexeeva *et al.*, 2010) |  |  |  |  |  |  |  |  |  |
| **pNM046** | pTHV037-mTq2-FtsZ | Cytoplasmic mTq2-FtsZ | This work | pSAV073 |  | NcoI | BsrGI | mTq2 | pNM059 | 234 + 86 | NcoI | BsrGI |
| **pNM047** | pTHV037-sfTq2-FtsZ | Cytoplasmic sfTq2-FtsZ | This work | pSAV073 |  | NcoI | BsrGI | sfTq2 | pNM067 | 234 + 86 | NcoI | BsrGI |
| **pNM048** | pTHV037-sfTq2^C70V^-FtsZ | Cytoplasmic sfTq2^ox^-FtsZ | This work |  |  |  |  |  |  |  |  |  |
| **pSAV089** |  | Used for cloning | (Alexeeva *et al.*, 2010) |  |  |  |  |  |  |  |  |  |
| **pNM049** | pSAV057-mTq2-FtsZ | Cytoplasmic mTq2-FtsZ | This work | pSAV089 |  | NcoI | BsrGI | mTq2 | pNM059 | 234 + 86 | NcoI | BsrGI |
| **pNM050** | pSAV057-sfTq2-FtsZ | Cytoplasmic sfTq2-FtsZ | This work | pSAV089 |  | NcoI | BsrGI | sfTq2 | pNM067 | 234 + 86 | NcoI | BsrGI |
| **pNM051** | pSAV057-sfTq2^C70V^-FtsZ | Cytoplasmic sfTq2^ox^-FtsZ | This work |  |  |  |  |  |  |  |  |  |
|  | **DsbA^ss^-PBP5 variants** |  |  |  |  |  |  |  |  |  |  |  |
| **C1-mOrange** | C1-mOrange | Used for cloning | (Kremers *et al.*, 2009) |  |  |  |  |  |  |  |  |  |
| **C1-mOrange2** | C1-mOrange2 | Used for cloning | (Kremers *et al.*, 2009) |  |  |  |  |  |  |  |  |  |
| **N1-mStrawberry** | N1-mStrawberry | Used for cloning | (Shaner *et al.*, 2004) |  |  |  |  |  |  |  |  |  |
| **C1-mScarlet** | C1-mScarlet | Used for cloning | (Bindels *et al.*, 2016) |  |  |  |  |  |  |  |  |  |
| **C1-mScarletI** | C1-mScarletI | Used for cloning | (Bindels *et al.*, 2016) |  |  |  |  |  |  |  |  |  |
| **C1-mScarletH** | C1-mScarletH | Used for cloning | (Bindels *et al.*, 2016) |  |  |  |  |  |  |  |  |  |
| **pNM118** | pTHV037-DsbA^ss^-LEGPAGL-sfGFP-GS-∆1-17-PBP5 | IM bound sfGFP-PBP5 | (Meiresonne *et al.*, 2017) | pNM12 |  | XhoI | BamHI | sfGFP |  | 150+151 | XhoI | BamHI |
| **pNM052** | pTHV037-DsbA^ss^-LEGPAGL-sfGFP^C70V^-GS-∆1-17-PBP5 | IM bound sfGFP^C70V^-PBP5 | This work |  | 236 + 237 |  |  |  |  |  |  |  |
| **pNM11** | pTHV037-DsbA^ss^-LEGPAGL-mNG^EC^-EFGS-∆1-17-PBP5 | IM bound mNG^EC^-PBP5 | (Meiresonne *et al.*, 2017) |  |  |  |  |  |  |  |  |  |
| **pNM053** | pTHV037-DsbA^ss^-LEGPAGL-mOr-EFGS-∆1-17-PBP5 | IM bound mOrange-PBP5 | This work | pNM12 |  | XhoI | BamHI | mOrange | C1-mOrange | 74+75 | XhoI | BamHI |
| **pNM054** | pTHV037-DsbA^ss^-LEGPAGL-mOr2-EFGS-∆1-17-PBP5 | IM bound mOrange2-PBP5 | This work | pNM12 |  | XhoI | BamHI | mOrange2 | C1-mOrange2 | 74+75 | XhoI | BamHI |
| **pNM055** | pTHV037-DsbA^ss^-LEGPAGL-mStr-EFGS-∆1-17-PBP5 | IM bound mStrawberry-PBP5 | This work | pNM12 |  | XhoI | BamHI | mStrawberry | C1-mStrawberry | 74+75 | XhoI | BamHI |
| **pNM12** | pTHV037-DsbA^ss^-LEGPAGL-mCh-EFSGRS-∆1-17-PBP5 | Used for cloning | (Meiresonne *et al.*, 2017) |  |  |  |  |  |  |  |  |  |
| **pNM056** | pTHV037-DsbA^ss^-LEGPAGL-mSc-EFSGRS-∆1-17-PBP5 | IM bound mScarlet-PBP5 | This work | pNM11 |  | XhoI | BamHI | mScarlet | C1-mScarlet | 73+74 | XhoI | BamHI |
| **pNM057** | pTHV037-DsbA^ss^-LEGPAGL-mScI-EFSGRS-∆1-17-PBP5 | IM bound mScarletI-PBP5 | This work | pNM11 |  | XhoI | BamHI | mScarletI | C1-mScarletI | 73+74 | XhoI | BamHI |
| **pNM058** | pTHV037-DsbA^ss^-LEGPAGL-mScH-EFSGRS-∆1-17-PBP5 | IM bound mScarletH-PBP5 | This work | pNM11 |  | XhoI | BamHI | mScarletH | C1-mScarletH | 73+74 | XhoI | BamHI |
|  | **monomeric turquoise2 variants in PBP5** |  |  |  |  |  |  |  |  |  |  |  |
| **pNM059** | pTHV037-DsbA^ss^-LEGPAGL-mTq2-EFGS-∆1-17-PBP5 | IM bound mTq2-PBP5 | This work | pNM12 |  | XhoI | BamHI | mTq2 | C1-mTq2 | 73+74 | XhoI | BamHI |
| **pNM060** | pTHV037-DsbA^ss^-LEGPAGL-mTq2^C48S^-EFGS-∆1-17-PBP5 | IM bound mTq2^C48S^-PBP5 | This work |  | 152 + 153 |  |  |  | pNM059 |  |  |  |
| **pNM061** | pTHV037-DsbA^ss^-LEGPAGL-mTq2^C70S^-EFGS-∆1-17-PBP5 | IM bound mTq2^C70S^-PBP5 | This work |  | 154 + 155 |  |  |  | pNM059 |  |  |  |
| **pNM062** | pTHV037-DsbA^ss^-LEGPAGL-mTq2^C70M^-EFGS-∆1-17-PBP5 | IM bound mTq2^C70M^-PBP5 | This work |  | 158 + 159 |  |  |  | pNM059 |  |  |  |
| **pNM063** | pTHV037-DsbA^ss^-LEGPAGL-mTq2^C48S-C70M^-EFGS-∆1-17-PBP5 | IM bound mTq2^C48S-C70M^-PBP5 | This work |  | 154 + 155 |  |  |  | pNM059 |  |  |  |
| **pNM064** | pTHV037-DsbA^ss^-LEGPAGL-mTq2^C48S-C70S^-EFGS-∆1-17-PBP5 | IM bound mTq2^C48S-C70S^-PBP5 | This work |  | 158 + 159 |  |  |  | pNM059 |  |  |  |
| **pNM065** | pTHV037-DsbA^ss^-LEGPAGL-mTq2^C48S-C70V^-EFGS-∆1-17-PBP5 | IM bound mTq2^C48S-C70V^-PBP5 | This work |  | 156 + 157 |  |  |  | pNM060 |  |  |  |
| **pNM066** | pTHV037-DsbA^ss^-LEGPAGL-mTq2^C48S-C70V-F99S^-EFGS-∆1-17-PBP5 | IM bound mTq2^C48S-C70V-F99S^-PBP5 | This work |  | 230 + 231 |  |  |  | pNM065 |  |  |  |
|  | **monomeric superfolder mTurquoise2 variants in PBP5** |  |  |  |  |  |  |  |  |  |  |  |
| **C1-sfTq2** | C1-sfTq2 | Maintenance plasmid, mTq2^S30R-Y39N-F99S-N105T-I171V^ |  |  |  |  |  |  |  |  |  |  |
| **pNM067** | pTHV037-DsbA^ss^-LEGPAGL-sfTq2-EFGS-∆1-17-PBP5 | IM bound sfTq2-PBP5 | This work | pNM12 |  | XhoI | BamHI | sfTq2 | C1-sfTq2 | 73+74 | XhoI | BamHI |
| **pNM068** | pTHV037-DsbA^ss^-LEGPAGL-sfTq2^C48S^-EFGS-∆1-17-PBP5 | IM bound sfTq2^C48S^-PBP5 | This work |  | 152 + 153 |  |  |  | pNM067 |  |  |  |
| **pNM069** | pTHV037-DsbA^ss^-LEGPAGL-sfTq2^C48V^-EFGS-∆1-17-PBP5 | IM bound sfTq2^C48V^-PBP5 | This work |  | 226 + 227 |  |  |  | pNM067 |  |  |  |
| **pNM070** | pTHV037-DsbA^ss^-LEGPAGL-sfTq2^C48R^-EFGS-∆1-17-PBP5 | IM bound sfTq2^C48R^-PBP5 | This work |  | 226 + 227 |  |  |  | pNM067 |  |  |  |
| **pNM071** | pTHV037-DsbA^ss^-LEGPAGL-sfTq2^C70A^-EFGS-∆1-17-PBP5 | IM bound sfTq2^C70A^-PBP5 | This work |  | 228 + 229 |  |  |  | pNM067 |  |  |  |
| **pNM072** | pTHV037-DsbA^ss^-LEGPAGL-sfTq2^C70G^-EFGS-∆1-17-PBP5 | IM bound sfTq2^C70G^-PBP5 | This work |  | 228 + 229 |  |  |  | pNM067 |  |  |  |
| **pNM073** | pTHV037-DsbA^ss^-LEGPAGL-sfTq2^C70M^-EFGS-∆1-17-PBP5 | IM bound sfTq2^C70M^-PBP5 | This work |  | 154 + 155 |  |  |  | pNM067 |  |  |  |
| **pNM074** | pTHV037-DsbA^ss^-LEGPAGL-sfTq2^C70P^-EFGS-∆1-17-PBP5 | IM bound sfTq2^C70P^-PBP5 | This work |  | 228 + 229 |  |  |  | pNM067 |  |  |  |
| **pNM075** | pTHV037-DsbA^ss^-LEGPAGL-sfTq2^C70S^-EFGS-∆1-17-PBP5 | IM bound sfTq2^C70S^-PBP5 | This work |  | 158 + 159 |  |  |  | pNM067 |  |  |  |
| **pNM076** | pTHV037-DsbA^ss^-LEGPAGL-sfTq2^C70T^-EFGS-∆1-17-PBP5 | IM bound sfTq2^C70T^-PBP5 | This work |  | 228 + 229 |  |  |  | pNM067 |  |  |  |
| **pNM077** | pTHV037-DsbA^ss^-LEGPAGL-sfTq2^C70V^-EFGS-∆1-17-PBP5 | IM bound sfTq2^ox^-PBP5 | This work |  | 156 + 157 |  |  |  | pNM067 |  |  |  |
| **pNM078** | pTHV037-DsbA^ss^-LEGPAGL-sfTq2^C48S-C70M^-EFGS-∆1-17-PBP5 | IM bound sfTq2^C48S-C70M^-PBP5 | This work |  | 154 + 155 |  |  |  | pNM068 |  |  |  |
| **pNM079** | pTHV037-DsbA^ss^-LEGPAGL-sfTq2^C48S-C70S^-EFGS-∆1-17-PBP5 | IM bound sfTq2^C48S-C70S^-PBP5 | This work |  | 158 + 159 |  |  |  | pNM068 |  |  |  |
| **pNM080** | pTHV037-DsbA^ss^-LEGPAGL-sfTq2^C48S-C70V^-EFGS-∆1-17-PBP5 | IM bound sfTq2^C48S-C70V^-PBP5 | This work |  | 156 + 157 |  |  |  | pNM068 |  |  |  |
| **pNM081** | pTHV037-DsbA^ss^-LEGPAGL-sfTq2^S99F^-EFGS-∆1-17-PBP5 | IM bound sfTq2^S99F^-PBP5 | This work |  | 232 + 233 |  |  |  | pNM067 |  |  |  |
| **pNM082** | pTHV037-DsbA^ss^-LEGPAGL-sfTq2^Y145F^-EFGS-∆1-17-PBP5 | IM bound sfTq2^Y145F^-PBP5 | This work |  | 238 + 239 |  |  |  | pNM067 |  |  |  |
| **pNM083** | pTHV037-DsbA^ss^-LEGPAGL-sfTq2^C70V-Y145F^-EFGS-∆1-17-PBP5 | IM bound sfTq2^C70V-Y145F^-PBP5 | This work |  | 238 + 239 |  |  |  | pNM077 |  |  |  |
|  | **Biological interaction, active/inactive PBP5 dimerization** |  |  |  |  |  |  |  |  |  |  |  |
| **pNM015** | pSAV057-DsbA^ss^-LEGPAGL-mNG^EC^-EFGS-∆1-17-PBP5-S44G | IM bound mNG^EC^-PBP5 S44G mutant | (Meiresonne *et al.*, 2017) |  |  |  |  |  |  |  |  |  |
| **pNM017** | pTHV037-DsbA^ss^-LEGPAGL-mNG^EC^-EFGS-∆1-17-PBP5-S44G | IM bound mNG^EC^-PBP5 S44G mutant | (Meiresonne *et al.*, 2017) |  |  |  |  |  |  |  |  |  |
| **pNM010** | pSAV057-DsbA^ss^-LEGPAGL-mCh-EFSGRS-∆1-17-PBP5 | Used for cloning | (Meiresonne *et al.*, 2017) |  |  |  |  |  |  |  |  |  |
| **pNM084** | pSAV057-DsbA^ss^-LEGPAGL-sfTq2-EFGS-∆1-17-PBP5 | IM bound sfTq2-PBP5 | This work | pNM10 |  |  |  |  | C1-sfTq2 | 75 + 74 | XhoI | BamHI |
| **pNM085** | pSAV057-DsbA^ss^-LEGPAGL-sfTq2-EFGS-∆1-17-PBP5-S44G | IM bound sfTq2-PBP5 S44G mutant | This work |  | 173 + 174 |  |  |  | pNM085 |  |  |  |
| **pNM086** | pTHV037-DsbA^ss^-LEGPAGL-sfTq2-EFGS-∆1-17-PBP5-S44G | IM bound sfTq2-PBP5 S44G mutant | This work |  | 173 + 174 |  |  |  | pNM067 |  |  |  |
|  | **OmpA variants** |  |  |  |  |  |  |  |  |  |  |  |
| **pNM014** | pSAV057-Ompa-(SA-1)-177-LEDPPAEL-mNG^EC^ | OM bound OmpA-mNG^EC^ reference | (Meiresonne *et al.*, 2017) |  |  |  |  |  |  |  |  |  |
| **pGV030** | pTHV037 OmpA-(SA-1)-177-LEDPPAEF-mCh | Used for cloning | (Verhoeven *et al.*, 2013) |  |  |  |  |  |  |  |  |  |
| **pNM004** | pTHV037-Ompa-(SA-1)-177-LEDPPAEL-mNG^EC^-mCh | Used for cloning | (Meiresonne *et al.*, 2017) |  |  |  |  |  |  |  |  |  |
| **pNM087** | pTHV037-Ompa-SA1-177-(SA-1)-LEDPPAEL-sfTq2 | OM bound OmpA-sfTq2 reference | This work | pGV030 |  | EcoRI | HindIII | sfTq2 | pNM067 | 39 + 86 | MfeI | HindIII |
| **pNM088** | pTHV037-Ompa-SA1-177-(SA-1)-LEDPPAEL-sfTq2^C70V^ | OM bound OmpA-sfTq2ox reference | This work | pGV030 |  | EcoRI | HindIII | sfTq2^ox^ | pNM077 | 39 + 86 | MfeI | HindIII |
| **pNM089** | pTHV037-Ompa-SA1-177-(SA-1)-LEDPPAEL-mNG^EC^-sfTq2 | OM bound OmpA-mNG^EC^-sfTq2 tandem | This work | pNM004 |  | EcoRI | HindIII | sfTq2 | pNM067 | 134 + 135 | EcoRI | HindIII |
| **pNM090** | pTHV037-Ompa-SA1-177-(SA-1)-LEDPPAEL-mNG^EC^-sfTq2^C48S^ | OM bound OmpA-mNG^EC^-sfTq2^C48S^ tandem | This work |  | 152 + 153 |  |  |  | pNM089 |  |  |  |
| **pNM091** | pTHV037-Ompa-SA1-177-(SA-1)-LEDPPAEL-mNG^EC^-sfTq2^C48S-C70V^ | OM bound OmpA-mNG^EC^-sfTq2^C48S-C70V^ tandem | This work |  | 156 + 157 |  |  |  | pNM090 |  |  |  |
| **pNM092** | pTHV037-Ompa-SA1-177-(SA-1)-LEDPPAEL-mNG^EC^-sfTq2^C70V^ | OM bound OmpA-mNG^EC^-sfTq2^ox^ tandem | This work | pNM004 |  | EcoRI | HindIII | sfTq2^ox^ | pNM077 | 39 + 86 | MfeI | HindIII |
| **pNM093** | pTHV037-Ompa-SA1-177-(SA-1)-LEDPPAEL-mNG^EC^-sfTq2^C70V^ | OM bound OmpA-mNG^EC^-sfTq2^ox^ tandem alternative | This work |  | 156 + 157 |  |  |  | pNM089 |  |  |  |
|  | **NlpA Variants** |  |  |  |  |  |  |  |  |  |  |  |
| **pNM094** | pTHV037-G-NlpA-GGS-mCh | IM bound NlpA-mCh | This work | pGV030 |  | NcoI | EcoRI | NlpA | LMC500 Chromosome | 165 + 172 | NcoI | MfeI |
| **pNM095** | pTHV037-G-NlpA-GGS-sfTq2 | IM bound NlpA-sfTq2 | This work | pNM087 |  | NcoI | EcoRI | NlpA | pNM094 | 165 + 172 | NcoI | MfeI |
| **pNM096** | pTHV037-G-NlpA-GGS-sfTq2^C70V^ | IM bound NlpA-sfTq2^ox^ | This work |  | 156 + 157 |  |  |  | pNM095 |  |  |  |
| **pNM097** | pSAV057-G-NlpAss-mNG^EC^ |  | Thiswork | pNM014 |  | NcoI | BamHi | NlpA^ss^ | pNM096 | 248 + 172 | NcoI | BamHI |
| **pNM098** | pTHV037-G-NlpA^SS^-KDPPAEL-mNG^EC^-sfTq2^C70V^ | IM bound NlpAss-mNG^EC^-sfTq2^ox^ tandem | This work | pNM093 |  | NcoI | BamHI | NlpA^ss^ | pNM096 | 248 + 172 | NcoI | BamHI |
|  | **LpoB Variants** |  |  |  |  |  |  |  |  |  |  |  |
| **pNM099** | pTHV037-G-LpoB-GGS-mCh | OM bound LpoB-mCh | This work | pGV030 |  | NcoI | EcoRI | LpoB | LMC500 Chromosome | 170 + 127 | NcoI | EcoRI |
| **pNM0100** | pTHV037-G-LpoB-GGS-sfTq2 | OM bound LpoB-sfTq2 | This work | pNM087 |  | NcoI | EcoRI | LpoB | LMC500 Chromosome | 170 + 127 | NcoI | EcoRI |
| **pNM101** | pTHV037-G-LpoB-GGS-sfTq2^C70V^ | OM bound LpoB-sfTq2^ox^ | This work |  | 156 + 157 |  |  |  | pNM100 |  |  |  |
| **pNM102** | pTHV037-G-LpoB73-mNG^EC^-sfTq2^C70V^ | OM bound LpoB73-mNG^EC^sfTq2^ox^ Tandem | This work | pNM093 |  | NcoI | BamHI | LpoB73 | pNM101 | 247 + 170 | NcoI | BamHI |
|  | **MalF Variants** |  |  |  |  |  |  |  |  |  |  |  |
| **pNM103** | pTHV037-MalF-^ss-mss^-mNG^EC^-sfTq2^C70V^ | IM bound MalFss-mss-mNG^EC^-sfTq2^ox^ tandem | This work | pNM100 |  | NcoI | BamHI | MalF^ss-mss^ | LMC500 Chromosome | 245 + 246 | NcoI | BamHI |
|  | **FtsB Variants** |  |  |  |  |  |  |  |  |  |  |  |
| **pNM104** | pTHV037-FtsB-sfTq2^C70V^ | IM bound FtsB-sfTq2^ox^ | This work | pNM088 |  |  |  | FtsB | LMC500 Chromosome | 249 + 250 | NcoI | BamHI |
| **pNM105** | pSAV057-FtsB-mNG^EC^ | IM bound FtsB-mNG^EC^ | This work | pNM014 |  |  |  | FtsB | LMC500 Chromosome | 249 + 250 | NcoI | BamHI |
| **pNM106** | pTHV037-FtsBm4-sfTq2^C70V^ | IM bound FtsB-sfTq2^ox^ Leucine Mutant | This work | pNM088 |  |  |  | FtsBmut4 | pCR124 | 249 + 250 | NcoI | BamHI |
| **pNM107** | pSAV057-FtsBm4-mNG^EC^ | IM bound FtsB-mNG^EC^ Leucine Mutant | This work | pNM014 |  |  |  | FtsBmut4 | pCR124 | 249 + 250 | NcoI | BamHI |
|  | **FtsL Variants** |  |  |  |  |  |  |  |  |  |  |  |
| **pNM108** | pTHV037-FtsL-sfTq2^C70V^ | IM bound FtsL-sfTq2^ox^ | This work | pNM088 |  |  |  | FtsL | LMC500 Chromosome | 251 + 252 | NcoI | BamHI |
| **pNM109** | pSAV057-FtsL-mNG^EC^ | IM bound FtsL-mNG^EC^ | This work | pNM014 |  |  |  | FtsL | LMC500 Chromosome | 251 + 252 | NcoI | BamHI |
| **pNM110** | pTHV037-FtsLm4-sfTq2^C70V^ | IM bound FtsL-sfTq2^ox^ Leucine Mutant | This work | pNM088 |  |  |  | FtsLmut4 | pCR119 | 251 + 252 | NcoI | BamHI |
| **pNM111** | pSAV057-FtsLm4-mNG^EC^ | IM bound FtsL-mNG^EC^ Leucine Mutant | This work | pNM014 |  |  |  | FtsLmut4 | pCR119 | 251 + 252 | NcoI | BamHI |
|  | **Eukaryotic constructs** |  |  |  |  |  |  |  |  |  |  |  |
| **N1-mCh** | N1-mCh | Used for cloning | (Shaner *et al.*, 2004) |  |  |  |  |  |  |  |  |  |
| **N1-mTq2** | N1-mTq2 | Maintenance plasmid | (Goedhart *et al.*, 2012) |  |  |  |  |  |  |  |  |  |
| **pNM112** | N1-sfTq2 | Maintenance plasmid | This work | N1-mCh |  | AgeI | BsrGI | sfTq2 | C1-sfTq2 | AgeI_mApple + 86 | AgeI | BsrGI |
| **pNM113** | N1-sfTq2^C70V^ | Maintenance plasmid | This work |  | 156 + 157 |  |  |  | pNM112 |  |  |  |
| **Cyterm-mKOκ** | Cyterm-mKOκ | Used for cloning | (Mastop *et al.*, 2017) |  |  |  |  |  |  |  |  |  |
| **Cyterm-mTq2** | Cyterm-mTq2 | For OSER asay | (Goedhart *et al.*, 2012) |  |  |  |  |  |  |  |  |  |
| **pNM114** | Cyterm-sfTq2 | For OSER asay | This work | Cyterm-mKOκ |  | AgeI | BsrGI | sfTq2 | C1-sfTq2 | AgeI_mApple + 86 | AgeI | BsrGI |
| **pNM115** | Cyterm-sfTq2^C70V^ | For OSER asay | This work |  | 156 + 157 |  |  |  | pNM114 |  |  |  |
| **SYFP2-T2A-SYFP2** | SYFP2-T2A-SYFP2 | Used for cloning | (Mastop *et al.*, 2017) |  |  |  |  |  |  |  |  |  |
| **SYFP2-T2A-mTq2** | SYFP2-T2A-mTq2 | For relative brightness assay | (Goedhart *et al.*, 2012) |  |  |  |  |  |  |  |  |  |
| **pNM116** | SYFP2-T2A-sfTq2 | For relative brightness assay | This work | SYFP2-T2A-SYFP2 |  | NotI | BamHI | sfTq2 | pNM087 | 241 + 242 | NotI | BamHI |
| **pNM117** | SYFP2-T2A-sfTq2^C70V^ | For relative brightness assay | This work | SYFP2-T2A-SYFP2 |  | NotI | BamHI | sfTq2^ox^ | pNM093 | 241 + 242 | NotI | BamHI |

# Table S4 – Primers used

| **Short Name** | **Long Name** | **Oligo sequence 5' → 3'** | **Additional information** |
| --- | --- | --- | --- |
| 39 | MfeI NeonGreenF | tttttt*caattg*ATGGTGAGCAAGGGCGA |  |
| 73 | 73-XhoI-link-NG | ggggg*ctcgag*ggtccggctggtctgATGGTGAGCAAGGGCGAG |  |
| 74 | 74-NG-nostop-BamHI | atatat*ggatcc*GAATTCCTTGTACAGCTCGTCC |  |
| 75 | 75-XhoI-link-mCherry | ggggg*ctcgag*ggtccggctggtctgATGGTGAGCAAGGGCGAGGAG |  |
| 86 | 86-NG-HindIII-R | gcgcgc*AAGCTT*TAGAATTCCTTGTACAGCT |  |
| 127 | 127-LpoB-nostop-EcoRI | ccccc*gaattc*gctaccaccTTGCTGCGAAACGGCACctttac |  |
| 134 | 134-EcoRI-FP | ccccc*gaattc*ATGGTGAGCAAGGGCGAGGAG |  |
| 135 | 135-FP-HindIII | ccccc*aagctt*CTACTTGTACAGCTCGTCC |  |
| 150 | 150-sfGFP-BamHI-nostop | gggggGGATCCTTTGTAGAGCTCATCCATGCCGTG |  |
| 151 | 151-XhoI-lnk-sfGFP | ggaaa*CTCGAG*GGTCCGGCTGGTCTGTCTAAAGGTGAAGAACTGTTCACCGG |  |
| 152 | 152-mTq2-C48S-F | CTGACCCTGAAGTTCATCT**c**CACCACCGG**t**AAGCTGCCCGTG | Introduces S48 in mTq2/sfTq2 and AgeI restriction site |
| 153 | 153-mTq2-C48S-R | CACGGGCAGCTT**a**CCGGTGGTG**g**AGATGAACTTCAGGGTCAG | “ |
| 154 | 154-mTq2-C70S-F | CACCCTGTCCTGGGG**t**GT**a**CAGT**c**CTTCGCCCGCTACCCC | Introduces S70 in mTq2/sfTq2 and BsrGI restriction site |
| 155 | 155-mTq2-C70S-R | GGGGTAGCGGGCGAAG**g**ACTG**t**AC**a**CCCCAGGACAGGGTG | “ |
| 156 | 156-mTq2-C70V-F | CACCCTGTCCTGGGG**t**GT**a**CAG**gt**CTTCGCCCGCTACCCC | Introduces V70 in mTq2/sfTq2 and BsrGI restriction site |
| 157 | 157-mTq2-C70V-R | GGGGTAGCGGGCGAAG**ac**CTG**t**AC**a**CCCCAGGACAGGGTG | “ |
| 158 | 158-mTq2-C70M-F | CACCCTGTCCTGGGG**t**GT**a**CAG**atg**TTCGCCCGCTACCCC | Introduces M70 in mTq2/sfTq2 and BsrGI restriction site |
| 159 | 159-mTq2-C70M-R | GGGGTAGCGGGCGAA**cat**CTG**t**AC**a**CCCCAGGACAGGGTG | “ |
| 165 | 165-NlpA-MfeI | ggaaa*caattg*gctaccaccCCAGCCAGGCACCGCGCCACCGTTAAA |  |
| 170 | 170-NcoI-G-LpoB | gggaa*ccATGg*gtACAAAAATGAGTCGCTACGCCTTGATTACCG |  |
| 172 | 172-NcoI-G-NlpA | gggaa*ccATGg*gtAAACTGACAACACATCATCTACGGACAGG |  |
| 173 | 173-PBP5-S44G-F | GATGTCCGCC**g**GGATCCTGCC**g**GCCTGACCAAAATGATGA | Introduces G44 in PBP5 and BamHI restriction site |
| 174 | 173-PBP5-S44G-R | TCATCATTTTGGTCAGGC**c**GGCAGGATC**c**CGGCGGACATC | “ |
| 226 | 226-mTq2-C48x-F | CTGACCCTGAAGTTCATC**nnk**ACCACCGGCAAGCTGCCCGTG | Introduces random mutation at position 48 of mTq2/sfTq2 |
| 227 | 227-mTq2-C48x-R | CACGGGCAGCTTGCCGGTGGT**mnn**GATGAACTTCAGGGTCAG | “ |
| 228 | 228-mTq2-C70x-F | CACCCTGTCCTGGGGCGTGCAG**nnk**TTCGCCCGCTACCCC | Introduces random mutation at position 70 of mTq2/sfTq2 |
| 229 | 229-mTq2-C70x-R | GGGGTAGCGGGCGAA**mnn**CTGCACGCCCCAGGACAGGGTG | “ |
| 230 | 230-mTq2-F99S-f | CCAGGAGCGCACCATC**agc**TTCAAGGACGACGGCaactac | Introduces S99 in mTq2 |
| 231 | 231-mTq2-F99S-r | gtagttGCCGTCGTCCTTGAA**gct**GATGGTGCGCTCCTGG | “ |
| 232 | 232-sfTq2-S99F | CCAGGAGCGCACCATC**tt**CTTCAAGGACGACGGCAcatac | Introduces F99 in sfTq2 |
| 233 | 233-sfTq2-S99F | gtatgTGCCGTCGTCCTTGAAG**aa**GATGGTGCGCTCCTGG | “ |
| 234 | 234-ncoi-*FP | ggggga*ccATGG*TGAGCAAGGGCGAG |  |
| 236 | 236-sfGFP-C70V-F | CCTGACCTATGGTGT**a**CAG**gt**TTTTTCTCGTTACCCGGATC | Introduces V70 in sfGFP and BsrGI restriction site |
| 237 | 237-sfGFP-C70V-R | GATCCGGGTAACGAGAAAAA**ac**CTG**t**ACACCATAGGTCAGG | “ |
| 238 | 238-sfTq2-Y145F-F | GGGGCACAAGCTGGAGTACAACT**t**CTTTAGCGACAACGTCTATATC | Introduces F145 in sfTq2 |
| 239 | 239-sfTq2-Y145F-F | GATATAGACGTTGTCGCTAAAG**a**AGTTGTACTCCAGCTTGTGCCCC | “ |
| 241 | 241-BamHI-mtq2 | cctgg*ggatcc*accggtcgccaccATGGTGAGCAAGGGCGAGGAGCTGTTCAC |  |
| 242 | 242-mtq2-not1 | gagtc*gcggccgc*tttaCTTGTACAGCTCGTCCATGCCGAGAGTGATC |  |
| 245 | 245-chrm-MalF-F | GCGTCGCATCAGGCAGTTGTTGTCGG | Binds upstream of *malF* |
| 246 | 246-MalF-ss-mss-BamHI-R | gaatat*ggatcc*tggccaTACATTAAAACAACAAGGTAACCCAC | Results in *malF* signal sequence and membrane spanning domain |
| 247 | 247-LpoB-trnc73-BamHI-R | gtatat*ggatcct*tCGGCGCAGGCGGTGCAGTTTGATCTTCG | Results in truncated *LpoB* at AA position 73 |
| 248 | 248-NlpA-trnc32-BamHI-R | gatata*ggatccT*TTGCATCGCTGCTACTCTGGTCGCAACCTGCCAG | Results in *nlpA* signal sequence |
| 249 | 249-NcoI-FtsB-F | gaatata*ccATGG*GTAAACTAACGCTGCTGTTGCTGGC |  |
| 250 | 250-FtsB-BamHI-R | gtatat*ggatcc*gcTCGATTGTTTTGCCCCGCAGACTG |  |
| 251 | 251-NcoI-G-FtsL | gtatat*ccATGg*gcATCAGCAGAGTGACAGAAGCTCTAAGC |  |
| 252 | 252-FtsL-BamHI-R | gtatat*ggatcc*gcTTTTTGCACTACGATATTTTCTTGTGACGG |  |
| AgeI_mApple | AgeI_mApple | gatcc*accggt*cgccaccATGGTGAGCAAGGGCGAGGAG |  |

Uppercase bases anneal to template DNA, *italics* signify restriction sites and **bold** underscores signify mutations. k = G or T, m= A or C, n = A,T,C or G.

# SI References

Alexeeva, S., Gadella, T.W.J., Verheul, J., Verhoeven, G.S., and Blaauwen, T. den (2010) Direct interactions of early and late assembling division proteins in Escherichia coli cells resolved by FRET. *Mol Microbiol* **77**: 384–98

Aronson, D.E., Costantini, L.M., and Snapp, E.L. (2011) Superfolder GFP is fluorescent in oxidizing environments when targeted via the Sec translocon. *Traffic* **12**: 543–8

Bindels, D.S., Haarbosch, L., Weeren, L. van, Postma, M., Wiese, K.E., Mastop, M., *et al.* (2016) mScarlet: a bright monomeric red fluorescent protein for cellular imaging. *Nat Methods* **14**: 53–56

Blaauwen, T. Den, Aarsman, M.E.G., Vischer, N.O.E., and Nanninga, N. (2003) Penicillin-binding protein PBP2 of Escherichia coli localizes preferentially in the lateral wall and at mid-cell in comparison with the old cell pole. *Mol Microbiol* **47**: 539–47

Costantini, L.M., Baloban, M., Markwardt, M.L., Rizzo, M., Guo, F., Verkhusha, V. V, and Snapp, E.L. (2015) A palette of fluorescent proteins optimized for diverse cellular environments. *Nat Commun* **6**: 7670

Cranfill, P.J., Sell, B.R., Baird, M.A., Allen, J.R., Lavagnino, Z., Gruiter, H.M. de, *et al.* (2016) Quantitative assessment of fluorescent proteins. *Nat Methods* **13**: 557–562

Dinh, T., and Bernhardt, T.G. (2011) Using superfolder green fluorescent protein for periplasmic protein localization studies. *J Bacteriol* **193**: 4984–7

Goedhart, J., Stetten, D. von, Noirclerc-Savoye, M., Lelimousin, M., Joosen, L., Hink, M.A., *et al.* (2012) Structure-guided evolution of cyan fluorescent proteins towards a quantum yield of 93%. *Nat Commun* **3**: 751

Kremers, G.J., Hazelwood, K.L., Murphy, C.S., Davidson, M.W., and Piston, D.W. (2009) Photoconversion in orange and red fluorescent proteins. *Nat Methods* **6**: 355–358.

Mastop, M., Bindels, D.S., Shaner, N.C., Postma, M., Gadella, T.W.J., and Goedhart, J. (2017) Characterization of a spectrally diverse set of fluorescent proteins as FRET acceptors for mTurquoise2. *Sci Rep* **7**: 11999

Meiresonne, N.Y., Ploeg, R. van der, Hink, M.A., and Blaauwen, T. den (2017) Activity-related conformational changes in D,D-carboxypeptidases revealed by in vivo periplasmic förster resonance energy transfer assay in escherichia coli. *MBio* **8**: e01089-17

Shaner, N.C., Campbell, R.E., Steinbach, P.A., Giepmans, B.N.G., Palmer, A.E., and Tsien, R.Y. (2004) Improved monomeric red, orange and yellow fluorescent proteins derived from Discosoma sp. red fluorescent protein. *Nat Biotechnol* **22**: 1567–72

Shaner, N.C., Lambert, G.G., Chammas, A., Ni, Y., Cranfill, P.J., Baird, M.A., *et al.* (2013) A bright monomeric green fluorescent protein derived from Branchiostoma lanceolatum. *Nat Methods* **10**: 407–9

Verhoeven, G.S., Dogterom, M., and Blaauwen, T. den (2013) Absence of long-range diffusion of OmpA in E. coli is not caused by its peptidoglycan binding domain. *BMC Microbiol* **13**: 66
